# Supplementary material for: Assessing the danger of self-sustained HIV epidemics in heterosexuals by population based phylogenetic cluster analysis
Source: eLife. 2017 Sep 12;6:e28721. doi: 10.7554/eLife.28721 (PMC5650480; doi:10.7554/eLife.28721)
Supplement: Transparent reporting form [file elife-28721-transrepform.pdf]

eLIFE
1st Floor
24 Hills Road
Cambridge CB2 1JP, UK
P 01223 855340
W elifesciences.org
T @elife

eLife's transparent reporting form

We encourage authors to provide detailed information *within their submission* to facilitate the interpretation and replication of experiments. If you have any questions, please contact us: editorial@elifesciences.org.

Sample-size estimation

- You should state whether an appropriate sample size was computed when the study was being designed
- You should state the statistical method of sample size computation and any required assumptions
- If no explicit power analysis was used, you should describe how you decided what sample (replicate) size (number) to use

Please outline where this information can be found within the submission (e.g., page numbers or figure legends), or explain why this information doesn't apply to your submission:

We used all the relevant available data from a cohort study, therefore a power analy sis was not performed. The details about the sample selection are explained on page 11 (Methods and Materials section).

Replicates

- You should report how often each experiment was performed
- You should include a definition of biological versus technical replication
- The data obtained should be provided and sufficient information should be provided to indicate the number of independent biological and/or technical replicates
- If you encountered any outliers, you should describe how these were handled
- Criteria for exclusion/inclusion of data should be clearly stated
- High-throughput sequence data should be uploaded before submission, with a private link for reviewers provided (these are available from both GEO and ArrayExpress)

Please outline where this information can be found within the submission (e.g., page numbers or figure legends), or explain why this information doesn't apply to your submission:

eLife Sciences Publications, Ltd is a limited liability non-profit non-stock corporation incorporated in the State of Delaware, USA, with company number 5030732, and is registered in the UK with company number FC030576 and branch number BR015634 at the address 1st Floor, 24 Hills Road, Cambridge CB2 1JP | August 2014

Since we did not perform any experiments and used only the existing data from an observational study, the number of replicates does not apply to our study.

The inclusion criteria of our sample are explained on page 11 (in the Phylogenetic tree section).

The HIV sequence data was obtained as part of the Swiss HIV Cohort Study (SHCS). Due to privacy reasons, the sensitivities associated with HIV infections, and the representativeness of the dataset, a deposition of the sequence data in an open database is not possible (Our data would in principle allow the reconstruction of transmission events and could thereby endanger the patients' privacy. This is especially problematic because HIV-1 sequences have been frequently used in court cases). From a scientific point of view, the consequences of an open and uncontrolled access to such densely sampled sequences could jeopardize the future publication (and thus the investigation) of similarly complete data-sets and thereby be contra-productive even from an "open-data" perspective. However, data can be made available for checking the results on a confidential basis and a sub-sample of 10% sequences from the SHCS has been uploaded to Genbank in the context of a previous publications. Moreover, all data in the SHCS can be used for well-defined projects that are in accordance with the guidelines of the SHCS, if a corresponding project proposal is approved by the SHCS scientific board.

Statistical reporting

- Statistical analysis methods should be described and justified
- Raw data should be presented in figures whenever informative to do so (typically when N per group is less than 10)
- For each experiment, you should identify the statistical tests used, exact values of N, definitions of center, methods of multiple test correction, and dispersion and precision measures (e.g., mean, median, SD, SEM, confidence intervals; and, for the major substantive results, a measure of effect size (e.g., Pearson's r, Cohen's d)
- Report exact p-values wherever possible alongside the summary statistics and 95% confidence intervals. These should be reported for all key questions and not only when the p-value is less than 0.05.

Please outline where this information can be found within the submission (e.g., page numbers or figure legends), or explain why this information doesn’t apply to your submission:

The statistical inference is described thoroughly in the Appendix 3 (p. 33—36). The p-values for key questions are provided in the main text (p. 4—5) and in Figure 3. All the results are graphically displayed together with the 95%-confidence intervals/bands.

eLife Sciences Publications, Ltd is a limited liability non-profit non-stock corporation incorporated in the State of Delaware, USA, with company number 5030732, and is registered in the UK with company number FC030576 and branch number BR015634 at the address 1st Floor, 24 Hills Road, Cambridge CB2 1JP | August 2014

(For large datasets, or papers with a very large number of statistical tests, you may upload a single table file with tests, Ns, etc., with reference to page numbers in the manuscript.)

Additional data files ("source data")

- We encourage you to upload relevant additional data files, such as numerical data that are represented as a graph in a figure, or as a summary table
- Where provided, these should be in the most useful format, and they can be uploaded as "Source data" files linked to a main figure or table
- Include model definition files including the full list of parameters used
- Include code used for data analysis (e.g., R, MatLab)
- Avoid stating that data files are "available upon request"

Numerical data for the Figure 1, upper panels of Figure 2 and Figure 3 are provided in the corresponding .csv files.

All the parameters used appear in Table 1 and are listed in Parameters_used_data.csv.

R package PoisTransCh which was implemented to analyse the data is attached as .tar.gz file. It can be also found on GitHub (https://github.com/tejaturk/PoisTransCh).

Please indicate the figures or tables for which source data files have been provided:
